# Conditions Associated with Marine Lipid-Induced Milk Fat Depression in Sheep Cause Shifts in the In Vitro Ruminal Metabolism of 1-^13^C Oleic Acid

**DOI:** 10.3390/ani8110196

**Published:** 2018-11-03

**Authors:** Pablo G. Toral, Gonzalo Hervás, Vanessa Peiró, Pilar Frutos

**Affiliations:** 1 Instituto de Ganadería de Montaña, CSIC-Universidad de León, Finca Marzanas s/n, 24346 Leon, Grulleros, Spain; pablo.toral@csic.es (P.G.T.); g.hervas@csic.es (G.H.); 2 Interdepartmental Research Service (SIdI), Autonomous University of Madrid (UAM), Calle Francisco Tomás y Valiente 7, 28049 Madrid, Spain; vanessa.peiro@uam.es

**Keywords:** ewe, lipid metabolism, oxygenated fatty acid, rumen, stable isotope, *trans* fatty acid

## Abstract

**Simple Summary:**

Dairy ewes, just like cows, suffer from the low-fat milk syndrome, also known as milk fat depression (MFD), when marine lipids are added to their diet to modulate milk fatty acid (FA) profile. Alterations in the ruminal metabolism of dietary unsaturated FA seem to be at the core of this MFD but its causative agent has not yet been confirmed. Although some alterations may derive from shifts in oleic acid metabolism, methodological constraints represent a major challenge: the fact that oleic and other dietary FA (e.g., linoleic and linolenic acids) have common intermediates hampers the identification of those metabolites deriving specifically from each one. Thus, we conducted an in vitro trial combining an isotopic tracer approach with the use of rumen inoculum from ewes fed a diet supplemented or not with fish oil (which is known to cause MFD in dairy animals), to provide new insights into shifts in oleic acid metabolism. Fish oil altered the relative contribution of specific pathways, with relevant increments in oxygenated FA, including a major candidate milk fat inhibitor (10-oxo-18:0). Changes in the concentration of *trans* 18:1 intermediates were also observed. Results are discussed in the context of their potential association with milk fat depression.

**Abstract:**

Shifts in ruminal oleic acid (OA) metabolism have received little research attention but recent studies have suggested their association with marine lipid-induced milk fat depression (MFD) in ewes and cows. Measurement of specific products of OA within the complex mixture of digesta lipids is however challenging. Therefore, this in vitro trial combined the isotopic labelling technique with the use of rumen inoculum from cannulated sheep fed a diet supplemented or not with 2% of fish oil (which has been demonstrated to cause MFD in dairy ruminants) to characterize the metabolism of OA in response to ruminal alterations associated with MFD. The products of ^13^C-OA after 24 h of incubation were analysed by gas chromatography-combustion isotope ratio mass spectrometry (GC-C-IRMS). Numerous ^13^C-labeled 18:1 intermediates and oxygenated FA were detected and no elongation or desaturation of ^13^OA occurred. Diet supplementation with fish oil (i.e., MFD conditions) resulted in no unique metabolites of ^13^OA but in relevant changes in the relative contribution of specific metabolic pathways. The inhibition of 18:0 saturation caused by this treatment appeared largely attributable to increased oxygenated FA proportion, in particular the candidate milk fat inhibitor 10-oxo-18:0, and warrants further research on the association between MFD and oxygenated FA. Changes in the concentration of ^13^C-labeled *trans* 18:1 intermediates but not in *cis* 18:1, were also observed.

## 1. Introduction

Milk fat depression (MFD) is a challenging biological problem characterized by a severe reduction of milk fat content, generally with no changes in milk yield or other components [1]. This reduction has been associated with the use of feeding strategies aimed at modulating milk fatty acid (FA) profile or sustaining high milk yields in dairy ruminants [2,3]. The economic value of milk fat and the associated losses during MFD justifies the emphasis laid on understanding the causes of this syndrome [3,4].

The MFD induced in cows by the consumption of plant oils or starch-rich diets, which is explained by shifts in ruminal biohydrogenation (BH) pathways that favour the accumulation of the antilipogenic *trans*-10 *cis*-12 conjugated linoleic acid (CLA) [2,3], is the most-widely studied MFD condition. However, although MFD induced by dietary marine lipid supplements was first described more than one century ago [1], its causative agent has not yet been confirmed [5,6]. Alterations of BH pathways are also presumed to be at the core of this type of MFD but extensive research has shown little or no change in *trans*-10 *cis*-12 CLA in response to fish oil and microalgae [2,7,8].

Marine lipid-induced changes in digesta FA profile are characterized by large decreases in 18:0 accumulation and opposite variations in most 18:1 and 18:2 intermediates and in some oxygenated FA [6,8,9]. The most prominent among them would be those occurring in recently proposed candidate milk fat inhibitors, such as *cis*-11 18:1, *trans*-10 *cis*-15 18:2 and 10-oxo-18:0 [6,9,10]. Some of these changes would be associated with shifts in the BH of α-linolenic acid, as reported in a recent in vitro study [11] but some others, particularly variations in 18:1 and oxygenated FA, may derive from alterations in the metabolism of oleic acid (OA) in the rumen [12,13]. In this regard, supplementation with OA-rich lipids has been reported to induce MFD in dairy cows [14,15,16] and, combined with fish oil, to decrease milk fat concentration and yield to a greater extent than a blend of this marine lipid and a source of α-linolenic acid [17]. Nevertheless, ruminal OA metabolism has received little attention, which might be due, at least to some extent, to methodological constraints: the fact that OA and other dietary unsaturated FA have common intermediates hampers the identification of those specifically derived from each one [3,18]. The isotopic labelling technique would offer a clear advantage to overcome this obstacle by measuring the products of a given tracer within the complex mixture of digesta lipids.

On this basis, we conducted an in vitro study with ^13^C-labeled OA to characterize its ruminal metabolism in ewes. Furthermore, we compared the use of rumen inocula from sheep fed a diet either without lipid supplementation or supplemented with 2% of fish oil (which has been demonstrated to decrease milk fat concentration by approximately 25% in this species) [6,19,20] with the aim of investigating the shifts in the ruminal metabolism of OA associated with marine lipid-induced MFD.

## 2. Materials and Methods

This experiment is an integral part of a larger study using stable isotopes (namely ^13^C-labeled FA) to provide new insights into ruminal BH. A first paper on the in vitro BH of ^13^C α-linolenic acid has recently been published [11] and contains detailed information about diets and rumen inocula composition that will therefore be only mentioned briefly in this new article.

### 2.1. Donor Animals

All protocols involving animals were approved by the Research Ethics Committee of the Instituto de Ganadería de Montaña (code 100102/2017-7, 8 January 2018), the Spanish National Research Council (CSIC; code 649/2018, 17 January 2018) and the Junta de Castilla y León (Spain), following proceedings described in Spanish and EU legislations (R.D. 53/2013 and Council Directive 2010/63/EU). All animals used in this study were handled in strict accordance with good clinical practices and all efforts were made to minimize suffering.

Five rumen-cannulated dry ewes (BW = 65.5 ± 2.28 kg) were used as rumen inocula donors for the batch cultures of microorganisms, which were conducted as described previously [11,21]. Sheep were randomly divided into two groups and fed a total mixed ration (TMR) with no lipid supplementation (to obtain the control inoculum; 3 ewes) or supplemented with 2% dry matter (DM) of fish oil (to obtain the FO-MFD inoculum; 2 ewes).

The basal TMR was formulated with a forage:concentrate ratio 50:50 from dehydrated alfalfa hay and a concentrate of whole corn and barley grains, soybean meal, sugar beet pulp, molasses and minerals and vitamins. It contained (on a DM basis) 18.7% of crude protein and 29.2% of neutral detergent fibre. In the supplemented diet, the fish oil (Afampes 121 DHA; Afamsa, Mos, Spain) replaced other dietary ingredients on a proportionate basis. The FA composition of both diets (% of total FA, data for control and supplemented TMR, respectively) included mainly 16:0 (22.6 vs. 21.7), *cis*-9 18:1 (14.8 vs. 14.5), 18:2n-6 (40.1 vs. 22.8) and 18:3n-3 (13.2 vs. 7.2), while EPA (3.3), DPA (0.7) and DHA (9.3) were only detected in the fish oil diet. Please see Toral et al. [11] for details.

Experimental diets were offered once daily at 1.2 times their estimated maintenance energy requirements [22]. Clean drinking water was always available.

### 2.2. Batch Cultures of Rumen Microorganisms

After sheep were fed the experimental diets for 21 days, we collected individual rumen fluids via the cannula before feeding. They were taken in thermal flasks to the laboratory and strained through a nylon membrane (250 µm; Fisher Scientific S.L., Madrid, Spain) under continuous flushing with CO_2_. Equal volumes of the strained rumen fluid obtained from each ewe were combined by treatments (i.e., control and FO-MFD) and used as the inoculum in the incubations.

The incubations were conducted in 16-mL Hungate tubes containing 12 mL of a mix (1:4) of the corresponding rumen inoculum and a phosphate-bicarbonate buffer [23]. An amount of 0.75 mg of ^13^OA ([1-^13^C] *cis*-9 18:1, 99% atom ^13^C%; CLM-149-PK, Cambridge Isotope Laboratories Inc., Tewksbury, MA, USA), equivalent to 1% of substrate DM, was dosed into each Hungate tube. To facilitate the dosing, this ^13^OA had been dissolved in hexane, the solution being added to the tubes and evaporated to dryness under a steady stream of nitrogen. Then, the basal diet fed to the ewes on the control treatment (ground in a hammer-mill fitted with a 0.5 mm screen) was used as the substrate and weighed into each tube (6.25 mg DM/mL of buffered rumen fluid). To determine the natural levels of ^13^FA in the digesta samples, we used another set of tubes with ^12^OA (≥99%; O1008; Sigma-Aldrich, Madrid, Spain) instead of ^13^OA, which was prepared following the same procedures.

The collection of the rumen inocula and the batch cultures were repeated on 3 different days (3 runs) and tubes with labelled and unlabelled OA were always incubated in duplicate under anaerobic conditions in an incubator set at 39.5 °C [2 inocula (control and FO-MFD) × 3 days × 2 OA (labelled and unlabelled) × 2 duplicates]. Incubations lasted for 24 h (when, according to previous preliminary assays, effects mimic those observed in vivo and were well detected) and the reaction was stopped by placing the tubes into ice-water for approximately 5 min. All the material was then frozen at −80 °C, freeze-dried and stored again at −80 °C until FA analysis.

### 2.3. Chemical Analyses

To determine the FA composition of the ruminal digesta in the Hungate tubes (which contained approx. 200 mg of freeze-dried digesta), we first extracted the lipids using 4 mL of a mixture of hexane and isopropanol (3:2, vol/vol) [24]. Extractions were repeated, and organic extracts were combined, dried at 45 °C under nitrogen and converted to FA methyl esters (FAME) by sequential base-acid catalysed transesterification [9]. Methyl esters were separated and quantified by GC-FID (Agilent 7890A GC System, Santa Clara, CA, USA) using a 100-m fused silica capillary column (CP-SIL 88, CP7489, Varian Ibérica S.A., Madrid, Spain) and hydrogen as the carrier gas. Total FAME profile was analysed using a temperature gradient program and then isothermal conditions at 170 °C to further resolve 18:1 isomers [24]. All peaks were identified based on retention time comparisons with commercially available FAME standards (from Sigma-Aldrich; Larodan, Solna, Sweden; and Nu-Chek Prep., Elysian, MN, USA), cross referencing with chromatograms reported in the literature [24,25] and comparison with reference ruminal digesta samples for which the FA composition was determined based on GC-FID analysis of FAME and gas chromatography-mass spectrometry analysis of corresponding 4,4-dimethyloxazoline derivatives [21,25].

Compound-specific isotope analysis (CSIA) was conducted using a gas chromatography-combustion isotope ratio mass spectrometry system (GC-C-IRMS; Thermo Scientific, Bremen, Germany) comprising a Trace GC Ultra gas chromatograph coupled to a Delta V Advantage isotope ratio mass spectrometer through a GC Combustion III interface maintained at 940 °C. To separate the FAME, we used the same CP-SIL 88 column and the isothermal program employed in GC-FID analysis, with helium as the carrier gas. The ^13^C% in the methanol employed for FA transesterification was determined with a FlashEA 1112-HT elemental analyser coupled to a Delta V Advantage isotope ratio mass spectrometer through a Conflo III interface (Thermo Scientific, Bremen, Germany). Methanol was placed in tin capsules and combustion was done at 1020 °C, with helium as the carrier gas.

### 2.4. Calculations and Statistical Analyses

The percentage of FA originating from the incubated ^13^OA was calculated as explained by Toral et al. [11]. First, the ^13^C% in the methanol was used to correct for the isotopic shift due to the extra methyl group in ^13^C-labeled FAME that were detected during GC-C-IRMS analysis. Second, to account for the natural levels of ^13^C, the ^13^C% of each FA measured in batch cultures with ^12^OA was subtracted from the respective ^13^C% in incubations with ^13^OA. For each fatty acid, the corrected ^13^C % determined by GC-C-IRMS and its relative proportion over total FA measured by GC-FID were used to determine the amount of the FA that would actually derive from the incubated ^13^OA.

The FA composition of ruminal digesta was analysed by a two-way ANOVA to test the fixed effects of inoculum type (control vs. FO-MFD), OA isotope (^12^OA vs. ^13^OA) and their interaction. For FA found exclusively in the FO-MFD treatment, the fixed effects due to OA isotope and inoculum by isotope interaction were removed. Enrichment data were analysed by a one-way ANOVA to test the fixed effect of the inoculum type. In both cases, the incubation run was designated as the random effect. Differences were declared significant at *p* < 0.05 and considered a trend towards significance at 0.05 ≤ *p* < 0.10. Statistical analyses were conducted using the SAS software package (version 9.4; SAS Institute Inc., Cary, NC, USA).

## 3. Results

### 3.1. Fatty Acid Composition of the Digesta

Inoculum type had a clear effect on the lipid composition of the digesta, whereas changes due to OA isotope were marginal and no significant differences were detected for the inoculum by isotope interaction (Table 1).

The large reduction in 18:0 concentration in the FO-MFD treatment (−47%; *p* < 0.001) was accompanied by significant increases in most 18:1 and 18:2 isomers, such as *cis*-11, *trans*-10 or *trans*-11 18:1 and *trans*-9 *cis*-12 or *trans*-11 *cis*-15 18:2. However, the concentration of *cis*-9 18:1 and 18:2n-6 were lower than in the control (−25% and −36%, respectively; *p* < 0.001). Differences due to the effect of FO-MFD inoculum were also found in the proportion of oxygenated FA, with a large increment in 10-oxo-18:0 and reductions in 13- and 16-oxo-18:0 (*p* < 0.05). Similarly, the profile of even-, odd- and branched-chain FA with less than 18 carbon atoms, such as 16:0, 17:0 or *iso* 17:0, was affected by inoculum type (*p* < 0.05). Very long-chain FA present in the fish oil and their BH intermediates were more abundant (*p* < 0.05) or could only be detected in FO-MFD treatment (e.g., total 20:1, EPA and DHA).

The few significant differences between incubations with ^12^OA and ^13^OA were limited to a lower proportion of OA in the latter (−12.5%; *p* < 0.001) and the opposite change in *trans*-13 + 14 and *trans*-15 18:1 and 9-oxo-18:0 + 10-OH-18:0 (*p* < 0.10). The concentration of EPA was marginally higher in incubations with ^13^OA (*p* < 0.05).

### 3.2. Ruminal Metabolism of ^13^OA

Table 2 shows the proportion of ^13^C-labeled FA detected in digesta at the end of the incubation, expressed as % of the initial ^13^OA. At that moment (end of incubation), the concentration of this isotopic tracer was lower in batch cultures with rumen fluid from ewes fed the fish oil diet than in the control, representing on average 12.7 and 17.2% of the incubated amount, respectively (*p* < 0.05). Therefore, a mean of 87.3 and 82.8% of the initial ^13^OA had disappeared in the FO-MFD and control treatments, respectively.

The major ^13^C-labeled product was always 18:0, although the FO-MFD treatment decreased its concentration by 16% (*p* < 0.05). The other 20 chromatographic peaks showing ^13^C enrichment over the natural levels were also identified as 18-carbon intermediates, specifically oxygenated FA and 18:1 isomers. No traces of labelling were detected in C18 PUFA or in FA with shorter or longer carbon chain length.

Among oxygenated FA, 10-oxo-18:0 was the most abundant product of ^13^OA, representing 2.2 and 14.7% of total ^13^C enrichment in the control and FO-MFD cultures, respectively (*p* < 0.05). The increase in the latter was accompanied by other relevant increments in 13- and 15-oxo-18:0 (*p* < 0.05), whereas 9-oxo-18:0 + 10-OH-18:0 remained unaffected by the fish oil supplementation (*p* > 0.10).

Inoculum type also had relevant effects on the profile of 18:1 isomers and the concentration of *trans* 18:1 isomers was generally greater in incubations with rumen fluid from ewes fed the fish oil diet. Significant increases ranged 50–63% for *trans*-6 + 7 + 8 to *trans*-12 18:1 intermediates and averaged 20% for *trans*-15 + *cis*-10 18:1 (*p* < 0.10). Conversely, the concentration of *trans*-16 + *cis*-14 18:1 decreased in FO-MFD incubations (*p* < 0.05). Other ^13^C-labeled 18:1 isomers (i.e., the abundant *trans*-13 + 14 18:1 and the minor *trans*-4, *trans*-5 and *cis* 18:1) were not affected by inoculum type (*p* > 0.10).

## 4. Discussion

Early investigations assumed that OA was metabolized in the rumen in a single step that led to 18:0 formation [26] but in vitro trials using isotopic tracers have shown that, under standard feeding conditions, OA metabolism yields numerous 18:1 intermediates [13,27]. However, no information was available on the fate of OA in response to the shifts in ruminal FA metabolism associated with marine lipid-induced MFD, which would be crucial to explain the origin of this syndrome [2,3,6]. Therefore, in this study we tried to mimic these alterations by incubating ^13^C-labeled OA with rumen inocula collected from ewes fed a diet supplemented with fish oil that was known to cause MFD in dairy ewes [6,19].

The diversity of ^13^OA products detected in our trial was greater than that in earlier studies using the same isotopic tracer, which described 18:1 intermediates with double bonds in *trans*-5 to *trans*-16 and in *cis*-11 to *cis*-16 positions, 10-OH-18:0 and 10-oxo-18:0 [12,13,28]. Compared with the usual GC-MS analysis, the greater sensitivity offered by the GC-C-IRMS methodology would be key to detect FA isotopes at low abundance levels [29] and, in our conditions, enabled the identification of some additional intermediates, such as *trans*-4 18:1, 13-oxo-18:0 and 15-oxo-18:0. The detection of ^13^C-labeled oxygenated FA was particularly important because they included a major metabolite (10-oxo-18:0) that accounted for almost 15% of the ^13^C enrichment in FO-MFD cultures. Remarkably, there is a scarcity of data in the literature about oxygenated FA of ruminal origin [10,21,30], which might be explained by their long retention times under standard chromatographic conditions and their consequent elution in less-well explored regions of the chromatogram [9]. For example, with a widely used 100-m polar column (CP-SIL 88) and the temperature gradient program described by Shingfield et al. [24], all oxo-18:0 metabolites eluted after 22:6n-3, the last of the very long chain PUFA that is usually quantified [5,9]. The fact the oxygenated FA are not usually present in feeds (please see Toral et al. [11] for a detailed FA composition of our diets) may also account for the limited research attention received by these products of ruminal microbiota [12,30].

Despite a thorough exploration of GC-C-IRMS chromatograms searching for traces of ^13^C enrichment, the FO-MFD treatment resulted in no unique metabolites but in relevant alterations in the accumulation of ^13^C-labeled FA. These findings are consistent with previous results from our trial examining the BH of ^13^C α-linolenic acid [11] and suggest that ruminal OA metabolism would follow the same pathways under standard (control) and FO-MFD conditions, although important differences in the relative contribution of each specific route exist.

Differences in the FA profile of digesta (Table 1) were consistent with other in vitro and in vivo trials describing the ruminal response to marine lipids [21,24,31]. Variations in some FA would be explained by their supply with the fish oil (e.g., 14:0, 16:1 or 20- and 22-carbon FA) [7,31] or by potential shifts in rumen microbial groups that synthesize them (e.g., odd- and branched-chain FA) [32]. Increases in conjugated and non-conjugated 18:2 intermediates, including some antilipogenic FA [2,3] would be common responses to marine lipids [8,31,33]. However, no 18:2 or 20- and 22-carbon FA was identified as a product of ^13^OA metabolism, supporting the hypothesis that rumen bacteria would not desaturate or elongate 18-carbon FA [11,34,35]. The lower concentration of 18:2n-6 in the FO-MFD treatment is consistent with the favourable effect of fish oil supplementation on the BH extent of dietary PUFA in the rumen [6,36,37].

Incubations with the FO-MFD inoculum enhanced OA disappearance too. The similarity of the reductions in OA proportion in ^13^C-labeled FA and in total FA in digesta (−26 and −25%, respectively) would indicate that fish oil had comparable effects on the metabolism of the ^12^C- and ^13^C-isotopes of OA, although some little differences have been reported (e.g., in *trans*-15 18:1 accumulation) [13]. The lack of variation in digesta FA (Table 1) due to the inoculum by isotope interaction further supports that the impact of fish oil would be similarly detected with both OA isotopes, which is crucial in our study.

The isotope analysis supports the hypothesis that marine lipids increase the extent of OA biohydrogenation [10,36], although some reports in the literature suggested no effects or even decreases in this parameter [31,37]. In this regard, it could be speculated that inconsistent results between studies not relying on isotopic tracer methodology could be accounted for by differences in dietary FA profiles, given that OA may be a BH intermediate of linoleic and linolenic acids [38,39]. Nevertheless, in the study by Toral et al. [11], the FO-MFD treatment also inhibited the accumulation of OA produced from the BH of ^13^C-linolenic acid. Other factors (e.g., ruminant species, interactions with basal diet composition or coelution with *trans* 18:1 in chromatography analysis) may then contribute to explain the erratic impact of marine lipids on ruminal OA proportion reported in the literature. Furthermore, the use of OA as a free FA or as part of triglycerides (i.e., readily available for microbial biohydrogenation or requiring previous lipolysis, respectively) [18] might also influence the response to marine lipids. In any event, the involvement of these changes in MFD might be limited, because the putative relevance of decreased OA availability for milk fat synthesis has been downplayed in dairy ewes [20].

Other effects of FO-MFD treatment on digesta composition differed between total and ^13^C-labeled FA (Table 1 and Table 2, respectively), which may help to improve our understanding of the complex BH of dietary FA [3,18]. Specifically, the comparison between changes in the concentration of ^13^C products and of total FA in digesta might help estimate the contribution of OA to ruminal alterations associated with marine lipid-induced MFD, including the production of candidate milk fat inhibitors [6,8,10].

For example, the lower decrease in the proportion of ^13^C-18:0 than of total 18:0 in digesta (−16 and −47%, respectively) suggests a lower contribution of OA metabolism to the inhibitory effect of marine lipids on the last BH step, compared with other dietary unsaturated FA such as linoleic and α-linolenic acids [10,11]. This might be explained, at least to some extent, by the putative high relevance of direct OA saturation to 18:0, without previous isomerization to *trans* 18:1 [18]. Marine lipids are strong inhibitors of *trans* 18:1 saturation in the rumen, probably through changes in microbial composition [31] but there is limited information on their impact on *cis* double bonds [11,21,33]. In any event, the decrease in ^13^C-18:0 proportion from 70.0 to 58.7% does not seem to be entirely attributable to greater *cis* and *trans* 18:1 accumulation. In fact, OA percentage was reduced in FO-MFD incubations, as discussed above, whereas other individual *cis* 18:1 isomers remained unaffected by inoculum type and total *trans* 18:1 concentration only increased 2.8 percentage points. Therefore, the key metabolites that appear to explain the shift in 18:0 production would be oxygenated FA, in particular 10-oxo-18:0, which increased from 2.2 to 14.7% of ^13^C-labeled FA.

Oxygenated FA seem to be final products of alternative FA metabolic pathways in the rumen, which involve sequential hydration and oxidation steps [12,40]. The rumen accumulation of 10-oxo-18:0 was previously related to OA input in the diet [12]. The greater proportion of this oxylipid in ^13^C-labeled products than in total FA in digesta, together with the similar fold-change due to FO-MFD treatment in both cases, supports that OA would be its main precursor. However, 13-oxo-18:0 and 15-oxo-18:0 were less abundant in ^13^OA products than in total FA in digesta and showed inconsistent responses to inoculum type, probably because they are major intermediates of other unsaturated FA, such as 18:2n-6 [40,41].

A potential association between 10-oxo-18:0 and marine lipid-induced MFD has been suggested in recent studies [5,20] but there is no conclusive evidence to confirm a cause and effect relationship. Because other oxylipids derived from unsaturated FA may have biological activity in dairy ruminants (e.g., 13-oxo-octadecadienoic and 5-hydroxy-eicosatetraenoic acids) [42], further research would be advisable to evaluate the effects of 10-oxo-18:0 on mammary lipogenesis. On the contrary, the association between 13- and 15-oxo-18:0 and MFD, if any, appears less relevant [5,6].

As mentioned above, variations in the proportion of ^13^C-labeled *cis* and *trans* 18:1 isomers were quantitatively lower than those described for oxygenated FA but some 18:1 isomers have biological effects and might be involved in marine lipid-induced MFD. For example, *cis*-11 18:1 has been proposed as a candidate milk fat inhibitor [5,19] based on its antilipogenic effect on bovine adipocytes [43]. However, its inconsistent variation in ^13^C-labeled FA and in total FA in digesta may indicate that that its increments are due to its presence in the fish oil and not to OA metabolism, in line with results from ^13^C α-linolenic acid incubation [11]. The similar lack of relationship for other *cis* 18:1 would also imply that OA did not contribute to their response to FO-MFD treatment.

On the other hand, our results would show that OA is a major precursor of some *trans* 18:1 intermediates regardless of inoculum type, such as *trans*-6 + 7 + 8, *trans*-9 and *trans*-13 + 14 18:1, which were less abundant in total digesta lipids than in ^13^OA products (Table 1 and Table 2, respectively), whereas the opposite was observed for *trans*-11 18:1. These results are in general agreement with previous studies using isotopic tracers [13,27] or oleic-rich lipid supplements [15,17,44]. However, although ^13^OA would not be a major precursor of *trans*-11 18:1, FO-MFD treatment favoured this BH pathway in the same proportion as that leading to *trans*-10 18:1 accumulation, which prevented a *trans*-11 to *trans*-10 18:1 shift in our conditions. A similar output was reported by Toral et al. [11]. In this regard, the relevance of the *trans*-11 to *trans*-10 shift as an effective marker of ruminal alterations associated with fish oil-induced milk fat depression seems minor in dairy ewes compared with cows [6,10,19].

## 5. Conclusions

Under the conditions of this research, the in vitro ruminal metabolism of ^13^OA yields numerous 18:1 intermediates, with double bonds in *trans*-4 to *trans*-16 and in *cis*-10 to *cis*-16 positions, and oxygenated products (9-oxo-18:0 + 10-OH-18:0 and 10-, 13- and 15-oxo-18:0). However, no desaturation to 18:2 or elongation to FA with 20 or 22 carbons takes place. Diet supplementation with fish oil (i.e., MFD conditions) results in no unique metabolites but in relevant changes in the relative contribution of specific metabolic pathways. The inhibition of 18:0 saturation caused by this treatment appears largely attributable to increased oxygenated FA proportion, in particular the candidate milk fat inhibitor 10-oxo-18:0, and warrants further research on the association between MFD and oxygenated FA. Changes in the concentration of ^13^C-labeled *trans* 18:1 intermediates but not in *cis* 18:1, are also observed.

## Figures and Tables

**Table 1:** Effects of rumen inoculum (control vs. FO-MFD ^1^) and oleic acid (OA) isotope (^12^C vs. ^13^C) on the fatty acid profile of digesta (expressed as % of total fatty acids) after 24 h of in vitro incubation.

Fatty Acid	Rumen Inoculum		OA Isotope				*p*-Value	
	Control	FO-MFD	^12^C	^13^C	SED ^2^	Inoculum	OA Isotope	Inoculum × OA Isotope
14:0	0.852	1.307	1.097	1.062	0.0324	<0.001	0.172	0.547
15:0	1.061	1.169	1.114	1.116	0.0190	<0.001	0.890	0.817
*anteiso* 15:0	0.593	0.474	0.530	0.536	0.0095	<0.001	0.448	0.333
16:0	12.338	17.653	15.014	14.978	0.2901	<0.001	0.864	0.944
∑ 16:1 isomers	0.094	0.332	0.214	0.212	0.0410	<0.001	0.960	0.884
17:0	0.802	0.939	0.871	0.870	0.0251	<0.001	0.945	0.871
*iso* 17:0	0.540	0.583	0.566	0.557	0.0223	0.035	0.606	0.531
*anteiso* 17:0	0.481	0.529	0.508	0.502	0.0086	<0.001	0.428	0.624
18:0	54.6	29.1	41.8	42.0	2.74	<0.001	0.929	0.880
Oxygenated fatty acids								
9-oxo-18:0 + 10-OH-18:0	0.021	0.029	0.021	0.029	0.0036	0.020	0.017	0.794
10-oxo-18:0	0.444	2.732	1.600	1.575	0.3503	<0.001	0.923	0.779
13-oxo-18:0	0.234	0.201	0.215	0.219	0.0190	0.041	0.778	0.886
15-oxo-18:0	0.037	0.042	0.038	0.041	0.0052	0.225	0.545	0.462
16-oxo-18:0	0.438	0.276	0.359	0.355	0.0142	<0.001	0.692	0.585
18:1 isomers								
*cis*-9 18:1	3.221	2.421	3.010	2.633	0.0908	<0.001	0.001	0.198
*cis*-11 18:1	0.161	0.325	0.234	0.252	0.0357	0.001	0.489	0.623
*cis*-12 18:1	0.268	0.254	0.256	0.267	0.0203	0.371	0.484	0.721
*cis*-13 18:1	0.053	0.100	0.075	0.078	0.0074	<0.001	0.604	0.499
*cis*-15 18:1	0.221	0.283	0.248	0.257	0.0169	0.002	0.471	0.606
*cis*-16 18:1	0.145	0.177	0.161	0.161	0.0086	0.002	0.927	0.906
*trans*-4 18:1	0.139	0.136	0.133	0.141	0.0137	0.777	0.420	0.420
*trans*-5 18:1	0.087	0.085	0.084	0.087	0.0074	0.703	0.637	0.558
*trans*-6 + 7 + 8 18:1	0.642	1.104	0.873	0.873	0.0413	<0.001	0.994	0.372
*trans*-9 18:1	0.454	0.912	0.677	0.689	0.0647	<0.001	0.801	0.607
*trans*-10 18:1	0.625	1.156	0.944	0.837	0.1156	0.001	0.239	0.733
*trans*-11 18:1	5.608	12.692	9.056	9.245	1.1404	<0.001	0.822	0.951
*trans*-12 18:1	0.752	1.532	1.118	1.165	0.0747	<0.001	0.410	0.753
*trans*-13 + 14 18:1	1.856	2.927	2.317	2.466	0.0942	<0.001	0.067	0.674
*trans*-15 + *cis*-10 18:1	0.916	1.289	1.006	1.199	0.0475	<0.001	0.001	0.241
*trans*-16 + *cis*-14 18:1	0.893	1.055	0.970	0.979	0.0661	0.014	0.853	0.882
*cis*-9 *cis*-12 18:2 ^3^	0.967	0.622	0.818	0.770	0.0695	<0.001	0.362	0.434
*trans*-9 *cis*-12 18:2	0.065	0.115	0.091	0.090	0.0108	0.001	0.882	0.480
*trans*-11 *cis*-15 18:2 ^4^	0.450	0.575	0.513	0.513	0.0473	0.010	0.992	0.945
*trans*-11 *trans*-15 + *trans*-9 *trans*-14 18:2	0.715	0.705	0.213	0.219	0.0220	0.001	0.715	0.705
*cis*-9 *trans*-11 18:2 ^5^	0.369	0.375	0.378	0.365	0.0271	0.764	0.530	0.552
*trans*-9 *cis*-11 18:2	0.038	0.057	0.047	0.048	0.0029	<0.001	0.686	0.205
*trans*-10 *cis*-12 18:2	0.035	0.063	0.047	0.051	0.0073	0.002	0.417	0.999
*trans*-11 *trans*-13 18:2	0.182	0.097	0.144	0.135	0.0154	<0.001	0.434	0.259
Other *trans*,*trans* 18:2 ^6^	0.353	0.247	0.299	0.301	0.0212	<0.001	0.897	0.359
*cis*-9 *cis*-12 *cis*-15 18:3 + *cis*-11 20:1	0.272	0.740	0.513	0.499	0.0475	<0.001	0.706	0.871
20:0	0.731	1.080	0.908	0.903	0.0465	<0.001	0.899	0.903
∑ 20:1 isomers	0.124	1.103	0.615	0.612	0.0551	<0.001	0.947	0.820
20:5n-3	-	0.043	0.041	0.044	0.0006	-	0.047	-
22:5n-3	-	0.031	0.031	0.030	0.0007	-	0.390	-
22:6n-3	-	0.055	0.057	0.054	0.0009	-	0.115	-

^1^ Derived from ewes fed a diet without lipid supplementation (control inoculum) or supplemented with 2% dry matter of fish oil (FO-MFD inoculum); ^2^ Standard error of the difference; ^3^ Contains *cis*-12 *cis*-16 and *trans*-12 *cis*-15 18:2 as minor components; ^4^ Contains *trans*-10 *cis*-15 18:2 as a minor component; ^5^ Contains *trans*-8 *cis*-10 18:2 as a minor component; ^6^ Sum of *trans*-7 *trans*-9 + *trans*-8 *trans*-10 + *trans*-9 *trans*-11 18:2.

**Table 2:** Effects of rumen inoculum (control vs. FO-MFD ^1^) on the proportion of ^13^C-labeled fatty acids in digesta (expressed as % of incubated ^13^C oleic acid) after 24 h of in vitro incubation.

^13^C-Labeled Fatty Acid	Control	FO-MFD	SED ^2^	*p*-Value
18:0	70.0	58.7	3.41	0.030
Oxygenated fatty acids				
9-oxo-18:0 + 10-OH-18:0	0.129	0.221	0.0580	0.186
10-oxo-18:0	2.186	14.658	3.3954	0.021
13-oxo-18:0	0.072	0.270	0.0249	0.001
15-oxo-18:0	0.016	0.069	0.0146	0.023
18:1 isomers				
*cis*-9 18:1	17.190	12.714	0.6419	0.020
*cis*-11 18:1	0.184	0.262	0.0313	0.130
*cis*-12 18:1	0.112	0.133	0.0207	0.401
*cis*-13 18:1	0.024	0.034	0.0048	0.105
*cis*-15 18:1	0.040	0.036	0.0200	0.845
*cis*-16 18:1	0.040	0.042	0.0082	0.867
*trans*-4 18:1	0.093	0.103	0.0491	0.849
*trans*-5 18:1	0.048	0.061	0.0318	0.708
*trans*-6 + 7 + 8 18:1	1.021	1.625	0.0805	0.002
*trans*-9 18:1	0.806	1.295	0.0380	0.006
*trans*-10 18:1	0.608	0.937	0.1155	0.047
*trans*-11 18:1	1.059	1.593	0.1548	0.026
*trans*-12 18:1	0.491	0.802	0.0210	0.005
*trans*-13 + 14 18:1	3.356	3.640	0.2320	0.346
*trans*-15 + *cis*-10 18:1	2.138	2.571	0.1910	0.086
*trans*-16 + *cis*-14 18:1	0.417	0.182	0.0207	0.008

^1^ Derived from ewes fed a diet without lipid supplementation (control inoculum) or supplemented with 2% dry matter of fish oil (FO-MFD inoculum); ^2^ Standard error of the difference.
